# Processing discharge summaries in general practice: a qualitative interview study with GPs and practice managers

**DOI:** 10.3399/bjgpopen18X101625

**Published:** 2019-01-23

**Authors:** Rachel A Spencer, Sarah Rodgers, Ndeshi Salema, Stephen M Campbell, Anthony J Avery

**Affiliations:** 1 NIHR Career Progression Fellow, Division of Primary Care, School of Medicine, University of Nottingham, Nottingham, UK; 2 NIHR Career Progression Fellow, Division of Primary Care, University of Warwick, Coventry, UK; 3 NIHR Career Progression Fellow, NIHR School for Primary Care Research, Oxford, UK; 4 Principal Research Fellow, Division of Primary Care, School of Medicine, University of Nottingham, Nottingham, UK; 5 Senior Research Fellow, Division of Primary Care, School of Medicine, University of Nottingham, Nottingham, UK; 6 Professor, Centre for Primary Care, University of Manchester, Manchester, UK; 7 Director, NIHR Greater Manchester Patient Safety Translational Research Centre, University of Manchester, Manchester, UK; 8 Professor, NIHR School for Primary Care Research, Oxford, UK; 9 Dean of the School of Medicine and Professor of Primary Health Care, Division of Primary Care, School of Medicine, University of Nottingham, Nottingham, UK; 10 Professor, NIHR School for Primary Care Research, Oxford, UK

**Keywords:** patient safety, education and standards, qualitative research, research methods, care of the elderly, patient groups

## Abstract

**Background:**

Discharge summaries are essential for communicating patient information from secondary care to general practice on hospital discharge. Although there has been extensive research into their design and completion in secondary care, very little is known about primary care processing of these documents.

**Aim:**

To explore what general practice staff think are the factors associated with failure to respond to actions requested in discharge summaries and what practices do to mitigate this.

**Design & setting:**

Semi-structured interviews were undertaken with primary care staff in three geographical regions of England.

**Method:**

Interviews with 10 practice managers and 10 GPs (one of each at each of the 10 practices) were undertaken to explore management of discharge summaries.

**Results:**

Five themes emerged from the interviews. The 'secondary care factors' theme describes participants’ perspectives on the design of summaries, which are inconsistent and often require improvement. The 'safety features of processing systems' theme focuses on document handling in primary care. A theme devoted to 'medicines reconciliation' followed. 'Error and harm as a result of faulty processing' is a theme describing ‘human error’ and other factors that participants believed contributed to failure to respond to requested actions. Finally, the 'strategies for safety improvement' theme describes initiatives to prevent failures of safer transitions of care.

**Conclusion:**

Correct processing of discharge summaries is essential to ensure patients experience a safe transition of care and not just a hospital discharge. Based on the interview findings, strategies to mitigate against faults in the processing of discharge summaries have been suggested to enhance safer transitions of care.

## How this fits in

All GP practices handle discharge summaries for older patients on a daily basis. The authors' recent paper in the British Journal of General Practice^1^ shows that discharge summary processing for this cohort is imperfect nearly half of the time, but little is known about why this might be the case. Therefore, the views of key staff in general practices were sought to discover what they believed were the reasons for incorrect processing and how this might be avoided. Based on the findings, a list of recommendations have been built for practices and hospitals to use to achieve safer care transitions.

## Introduction

The importance of high-quality safe care transitions, especially for older patients, is well described by public bodies.^2,3^ Unfortunately, care transitions are not always ideal: one in every five emergency admission patients has been discharged in the previous 48 hours.^4^ Healthwatch emphasises the importance of support continuing after the discharge event^5^ and, given the correct communication, general practice can facilitate this support. Discharge summaries, which are an essential link in the care transition information flow chain, have been intensively researched in secondary care,^6^ but little is known about how they are processed in primary care. Patients are potentially at risk of medications errors and lack of necessary follow-up should discharge summaries fail to be correctly processed. Error reports about discharge processes submitted by GPs to the National Reporting and Learning Service show that over three-quarters of patients involved in these reports had been harmed, but most of these reports focused on secondary care errors.^7^ Previous qualitative research relevant to discharge summaries in primary care has focused on discharge summary design^8^ rather than perceptions of the problems that occur within primary care systems. Previous work studying information flow from secondary to primary care identified common features of GP systems and highlighted the need for further investigation.^9^ As the burden of administrative workload in general practice increases (including potentially inappropriate delegation from secondary care), the challenge is to process this work safely. There is a paucity of specific guidelines on how to do this (although toolkits and care bundles addressing care transitions do exist).^10,11^

The qualitative work presented here was part of a wider mixed-methods study showing that failures occur in the processing of almost half of discharge summaries.^1^ The research question was: what do general practice staff think are the factors associated with failure to respond to actions requested in discharge summaries and what can practices do to mitigate this?

## Method

This was a qualitative semi-structured interview study with general practice staff using framework analysis. The mixed-methods approach to the research problem necessitated a practical approach to qualitative epistemology.^12^ The principles of social constructivism were followed, which allowed healthcare staff to share their stories of ‘why’ they do not adhere to instructions ‘given’ to them. At each site the lead researcher began by scrutinising in detail the information flow systems in operation by observing administrative staff handling paper and electronic mail, including discharge summaries (a case-study approach).^13^ For this reason, administrative staff perspectives were not sought again at interview. Questions about high-level procedural operations were also considered more suited to managerial level interviewees. Field notes were used to construct flow diagrams of each individual practice's system, using techniques adapted from healthcare failure modes effects analysis.^14^ To facilitate discussion, these diagrams were sent to participants some weeks before the interview, along with quantitative data^1^ and case vignettes of any harms detected.^1^

Interviews were conducted at the participating general practices in three areas of England. Ten practices were purposively sampled for the study^1^ from volunteer sites in the East Midlands, West Midlands, and in the North West to include a range of list sizes and different clinical information technology systems. Participants (senior GPs and practice managers from those 10 practices) were individually consented and none dropped out of the study.

Interviews were digitally recorded and followed the semi-structured interview guide, which was subject to minor adaptions as part of a reflexive process as the schedule of interviews progressed (further information available from the authors on request). Password-protected audio files were professionally transcribed. To ensure accuracy of transcription, all were checked by the lead researcher, following best practices for framework analysis.^15^ Transcripts were anonymised and emailed to participants for them to check. Coding was managed in NVivo (version 10). The framework was developed by the lead researcher in an iterative process, as described in worked examples provided in framework methodology literature.^15^ A code book was subsequently developed by two members of the practice team. Quality of coding was checked at a face-to-face meeting with co-authors with qualitative expertise.

## Results

Twenty interviews (of 29–62 minutes duration) were conducted. Descriptions of the practices and participant demographics are shown in Box 1 and Table 1 respectively.

**Box 1.** Descriptions of participating general practices

Practice number	Description
1	An urban training practice in the East Midlands with an average list size using SystmOne
2	A suburban training practice in the East Midlands with a larger than average list size using SystmOne
3	A rural training practice in an affluent (IMD <10) area of the East Midlands, with a larger than average list size using SystmOne
4	An urban training practice in a socioeconomically deprived (IMD >40) area of the East Midlands with an average list size using Emis Web
5	A suburban practice in a socioeconomically deprived (IMD >40) area of the East Midlands with an average list size using SystmOne
6	An urban training practice in a socioeconomically deprived (IMD >40) area of the West Midlands with a smaller than average list size using Emis Web
7	An urban training practice in the West Midlands with an average list size using SystmOne
8	A suburban practice in the East Midlands with an average list size using SystmOne
9	A suburban training practice in the North West with a smaller than average list size using Emis Web
10	An urban training practice a socioeconomically deprived (IMD >40) area of the North West. ‘Super-surgery’ (list size >20 000) using Emis Web

Practice 1 is the working site of GP1 and practice manager 1, practice 2 is the working site of GP2 and PM2, and so on. Where not stated, the practice had average IMD scores. Practices have been anonymised and randomly numbered to protect the identity of interview participants. IMD = index of multiple deprivation.

**Table 1.** Participant demographics

Participant code	Age, years	Sex	UK graduate(GP)	Graduation date	Years as qualified GP	Years in current profession (PM)	Date interviewed	Interview duration, mins
GP1	56	M	Yes	1984	25	NA	24/05/2017	54
GP2	38	F	No	2000	6	NA	22/11/2016	42
GP3	38	M	Yes	2004	6	NA	11/01/2017	39
GP4	38	F	Yes	2003	8	NA	16/11/2016	39
GP5	52	M	Yes	1988	24	NA	08/12/2016	62
GP6	38	M	No	2000	9	NA	10/05/2017	53
GP7	39	F	Yes	2004	9	NA	01/03/2017	33
GP8	49	F	Yes	1991	22	NA	25/01/2017	29
GP9	36	F	Yes	2006	5	NA	09/03/2017	48
GP10	60	M	Yes	1982	25	NA	19/04/2017	48
PM1	61	F	NA	NA	NA	23	22/6/2016	36
APM2	28	F	NA	NA	NA	1	22/11/2016	34
APM3	59	F	NA	NA	NA	20	11/01/2017	38
PM4	50	F	NA	NA	NA	25	16/11/2016	30
APM5	55	F	NA	NA	NA	37	08/12/2016	43
PM6	67	F	NA	NA	NA	15	10/05/2017	54
PM7	53	M	NA	NA	NA	2	01/03/2017	43
PM8	61	F	NA	NA	NA	9	25/01/2017	37
PM9	52	F	NA	NA	NA	8	09/03/2017	39
APM10	45	M	NA	NA	NA	22	19/04/2017	35

Practice 1 is the working site of GP1 and practice manager 1, practice 2 is the working site of GP2 and PM2, and so on.

APM = assistant practice manager. NA = not applicable. PM = practice manager.

Five main themes emerged from the interviews; these are detailed, along with their subthemes, in Box 2. The order of the themes follows the journey of the discharge summary document, from creation in secondary care through primary care administrative systems, to clinical processing (where error or harm might occur), and finally to strategies for safety improvement, where system redesign in response to these errors has happened. Most themes map closely to the original semi-structured interview schedule, but ‘secondary care factors’ emerged de novo. Some minor subthemes are not reported on here owing to word limit constraints (see Box 2 subthemes in brackets).

**Box 2.** Main themes and subthemes (subthemes in brackets are not reported on in this article)

1. **Secondary care factors**: ‘ideal’ discharge summary, delegation, (safety of transfer and NHS pressures) 2. **Safety features of discharge summary processing systems**: continuity of care, (team-working), protocols and procedures, training, staff numbers, and workflow targets 3. **Medicines reconciliation**: communication with patients, (medicine and/or patient related factors), pharmacy intervention, and prescribing roles 4. **Error and harm as a result of faulty processing of discharge summaries**: causes of error and/or harm, time and/or workload pressures, communication about error and/or harm, and harm severity and/or preventability 5. **Strategies for safety improvement**: administrative, IT and prescribing systems, significant event analyses, (the unplanned admissions scheme), and (direct impacts of the project)

**Box 3.** Recommendations for safer processing of discharge summaries

**Discharge summary design** Standardised structure ‘GP action' box Alphabetisation of medications listing Highlight changed medication (for example by using standard codes) Do not allow ‘hand annotations’ **Primary care administration team** On-the-day workflow system for electronic documents Priority scanning for paper discharge summaries Priority flag discharge summaries within the electronic mailbox Protected time and workspace for administrators involved in processing discharge summaries Aim for usual or referring GP to process the discharge summary unless this will lead to potentially hazardous delay **Primary care clinical team (or trained administrative personnel)** Prioritise higher-risk patients and/or carers to contact for medications reconciliation Consider making free-text entries about clinical decisions taken after reading the discharge summary (this could be recorded at the place where the discharge summary document can be seen in the facing electronic record) Consider using a clinical pharmacist to assist with medicines reconciliation Use IT to track actions; for example, electronic tasks, coded actions, diary functions Protected time and workspace for GPs and other colleagues involved in taking action in relation to requests in discharge summaries **Wider systems redesign** Enable visualisation of discharge summary and electronic health record at same time (split screen or two screens) Consider creating a template or consultation style in the electronic record (where possible, Read coded) to process the discharge summary; for example, data-entry points for diagnosis, significant test results, medication changes, discussions with patients and/or carers, and outstanding actions Interoperability of primary and secondary care IT systems to allow for co-creation of the discharge summary. GPs would be able to comment on medication changes and requests for action prior to the patient being discharged

### Secondary care factors

Despite secondary care issues not being directly raised by the interviewer, it was clear that GPs, in particular, wanted to highlight some of their frustrations with discharge summaries and make suggestions for improvement. GPs felt strongly about delegation of workload from secondary to primary care. Delegation (of referrals and tests) was cited in four harm cases discussed during the interviews. Most GPs reluctantly accepted delegation as part of their working lives, sometimes citing that they might be the best placed clinician to undertake the actions requested of them:

*'Two people who* [request] *loads of actions for us … that is the renal physicians and the falls team. Okay, so there is always like five actions every time they write to you but at least you know that and you know there is a section that says GP action, okay and so you go: "Oh god another one of these, okay let’s do all of their actions for them."’* (GP1, 56 year old male)

A few GPs were active in their rebuttal of work, particularly chasing test results, but admitted that this probably took up more of their time than just completing the required activity. There was concern among practice managers that poor understanding of healthcare finances might lead to inappropriate delegation:

*'They are very quick to throw it to primary care without even understanding what costings we have in our budgets to actually do some of the things they request us to do and I think that is where some of the problems lie.'* (PM9, 52 year old female)

Standardisation of the structure of a discharge summary was highlighted as an important issue with emphasis on clarity and the need for a ‘GP action’ box:

*Depending on how they’re written, there should be a GP action point at the bottom … that would be the best place to put things, not in the ‘this is what happened’, big paragraph at the front.'* (GP8, 49 year old female)

The accuracy of the medications reconciliation part of the discharge summary was a recurrent frustration, even in the East Midlands where substantial efforts had been made to standardise it by assigning codes to medications to indicate whether medications are new, changed, or unchanged: ^16^

*'Having the thing in the medication where there was something new or added or changed, that was quite helpful, certainly people can then pick it up from there but you have a degree of scepticism to make sure you have checked everything else as well because they can get it wrong sometimes.'* (GP2, 38 year old female)

The accuracy of the medications reconciliation section of the summary appeared worse in the other geographical regions, especially in the West Midlands, where there was a particular problem with the pharmacist’s hand annotations in coloured pen, which frequently scanned poorly:

*Sometimes the discharge letter tells you ‘new’ tablets … but it doesn’t sort of say stop other ones that they might already be on … Sometimes you can’t read them, if they are written they are absolutely horrendous.* (PM6, 67 year old female)

The simple intervention or alphabetisation of the medications reconciliation part of the summary was mentioned by several participants. This would then place the drugs in the same order as that occurring on the GP's system.

### Safety features of discharge summary processing systems

Continuity of care emerged as a major subtheme in relation to the systems practices had for processing discharge summaries, where sending the summary to the ‘right’ clinician was an integral part of the process. Clinicians felt they could offer an enhanced service for patients they knew better, expressing personal pride and professional curiosity in following up certain patients, particularly when significant diagnoses had been made:

*'I just kind of see the names, if I recognise them it makes it so much easier … I would give them a call, especially if knew them or whoever had been looking after them, I would say we need to call this person because they have developed cancer or whatever.'* (GP7, 39 year old female)

A trade-off was described between getting the ‘right’ clinician for the summary versus the need to process it in a timely fashion. Four of the 10 practices had systems that ensured discharge summaries were processed by clinicians on the day of arrival. In the other practices, participants felt that a week was a reasonable target for clinical processing. Managers described difficulties with getting the document to the ‘usual’ or referring clinician, but felt the effort was required for better patient care:

*'It* [work-flowing the summary] *feels such a trivial … well it is not trivial because it should go back to the person that referred them, but you know sometimes trying to unpick all of that is an onerous task.'* (PM8, 61 year old female)

### Medicines reconciliation

Medicines reconciliation (defined by the Institute for Healthcare Improvement as 'the process of creating the most accurate list possible of all medications a patient is taking')^17^ is a well-defined strategy to improve patient safety at discharge from hospital,^18^ and guidance for general practice does exist.^19^ Some responders felt it was impracticable to discuss medicines reconciliation with patients or carers following hospital discharge, but frequently expressed hope that it had been done in hospital:

*'Well I would hope that the hospital have discussed their medicine changes if they initially prescribed them … not just wandered around randomly giving them something to take or something taken off.'* (GP8, 49 year old female)

At the same time, participants identified problems with this approach, particularly in relation to vulnerable patients and their carers. Time was cited as a barrier to calling patients about medicines reconciliation, but the majority view was that this would be desirable:

*I think ideally that* [calling patients] *would be great … that is an additional workload … it is a definite positive if it was feasible to do it … for a lot of people it probably would either be a reassurance or flag up if they are aware of what the change has been from hospital.* (GP4, 38 year old female)

The prevailing view of practice managers was that medicines reconciliation was outside the role of practice receptionists, although participants did acknowledge that, with appropriate training, prescriptions clerks could undertake this role:

*'It is an awful risk isn’t it? Putting new medication on? You have no idea what these things are for or … any dangers about dosage or, you know, no it is absolutely a no-no ... we decided doctors put it on, because … if they have got to check it, they may as well do it.'* (PM8, 61 year old female)

One practice had already invested heavily in training their receptionists to manage medicines reconciliation, but the GP described some resistance to the changes:

*'What we keep telling* [them]*, because some of them were reluctant to make changes, is that the final responsibility lies with the GP so you know nothing gets filed until we sign it off so if there is any medication errors we should be double-checking … there is always the safety net.'* (GP3, 38 year old male)

Participants were generally positive about having a pharmacist in practice to perform medicines reconciliation, but most cited lack of funding as a major barrier:

*'It would be a very useful role but it would cost the same as having another doctor and another doctor would probably be more useful.'* (GP1, 56 year old male)

### Error and harm as a result of faulty processing of discharge summaries

Participants described a number of interrelated active and latent failures^20^ across a broad range of activities.^21^ For example, distraction as an element of slips and lapses was described, alongside time and workload pressures in the working environment:

*It is not an ideal world and things do … either get missed or I am going to do that in a minute and then something else happens and … you know you sort of forget about what you were going to deal with because something else has cropped up.* (PM6, 67 year old female)

GPs described slips and lapses occurring owing to overfamiliarity with the work:

*'We become a bit snow blind that we sort of read of "Oh yes somebody in with an infection exacerbated their COPD and blah, blah, blah. P.S. They were in acute renal failure please check their U&Es* [urea and electrolytes] *again."’* (GP5, 52 year old male)

Slips and lapses were largely ascribed to ‘human nature’ and were accepted as part of life by the majority:

*'You have got like 130 to go through, some people may not be as careful as others and that is human.'* (APM3, 59 year old female)

The majority of practices did not have dedicated time for GPs to do administrative work, necessitating GPs to process summaries in what was already a very full day, taking advantage of *'the squeezed in-between time'* (GP4, 38 year old female), or resorting to after-hours working:

*I have a laptop that has access to the medical record which I take home, so nine o’clock at night, ten o’clock at night, I do it.* (GP5, 52 year old male)

Many GPs described acceptance of the increasing problems of trying to keep on top of their workload:

*'I used to love trying to clear my inbox but I just accepted I am never going to do it … we have sort of a warning system where we have to clear them within so many days so I get those done and the rest can wait until the next tranche, you know.'* (GP10, 60 year old male)

The mismatch between Electronic Document Management Systems (EDMS) and clinical IT systems was often implicated as contributing to errors:

*'It is sometimes difficult on a, you know, electronic format that you’re flitting between screens trying to see what is going on and you sometimes will miss stuff.'* (GP5, 52 year old male)

### Strategies for safety improvement

Opinion on which personnel should process summaries was divided. Some were vehemently against the extension of administrative staff roles owing to safety concerns stating that *'Important things like discharge letters should be scanned to the GP,'* (GP8, 49 year old female) while others could see time and efficiency savings from engaging administrative staff but still had concerns about safety, duplication of workload, medicolegal issues, and training:

*'I think that would need protected time as well so I don’t think I would mind it if they could dedicate their full attention to it but definitely not as a sideline, as they are answering the phones and on reception.'* (APM2, 28 year old female)

Methods to improve discharge summary processing suggested by participants included: priority scanning, priority marking in the EDMS, and creation of an on-the-day workflow. Although clinical IT was reported as having potential to improve the processing of summaries, two practices had reverted to paper systems for discharge summaries specifically, and many GPs were printing summaries in order to reconcile medications. Electronic ‘task’ systems were both the solution to, and the cause of, problems with summary processing.

Read-coding actions was a strategy at one practice, which ran daily searches for codes (such as requirement for blood tests), which were inputted by their doctors when processing a summary. The administrative team at another practice used the ‘diary’ function of their clinical systems in a similar manner, checking for completed actions regularly and sending them back to clinicians. Using IT strategies to improve medicines reconciliation was considered desirable, as was alignment of primary and secondary care IT systems. Positive experiences of the Salford integrated care record^22^ were described by some participants:

*'I had access to those notes too so I could snoop and say right … Well what have the hospital done? And then I could actually input on to the notes "Look I don’t think you should start the patient on this, I have tried it before, they had this reaction, X, Y, Z" and not only could I add my piece but … when they come out I could see why certain changes had been made.'* (GP9, 36 year old female)

## Discussion

### Summary

Twenty in-depth interviews were conducted with senior general practice staff in three different parts of England on a novel topic. Participants identified concerns with design and completion of discharge summaries. They offered reasons for incorrect processing in general practice and described strategies they had used to mitigate against this. Recommendations for safer management based on the findings are described in Box 3.

### Strengths and limitations

Using a single interviewer helped to ensure consistency; the lead researcher had already investigated the practices’ systems and was able to approach the interviews fully informed of this information. The team approach to the interview schedule design and subsequent quality-checking during the analysis of data served to minimise bias. A GP interviewer and the lack of anonymity could be seen as limitations but, as the results suggest, practice staff were able to speak candidly about a taboo subject. Indeed, participants may have been more welcoming of analysis of their highly personal systems by a perceived ‘insider’. The occurrence of de novo themes and the presence of divergent views are indicative of theoretical saturation that allowed a narrative to be developed. The in-depth interviews were adequate in length and conducted with appropriate participants from a culturally cohesive sample. Thematic saturation has been achieved, evidenced by no new ideas emerging toward the end of the interview (except in the ‘improvement strategies’ theme, where it was not anticipated).

### Comparison with existing literature

Participants felt that electronic discharge summary design had scope for improvement, echoing previous findings.^21^ Delegation of workload to primary care remains a contentious issue, as demonstrated in other primary care provider interviews^23,24^ and, despite guidelines to the contrary,^25^ some participants felt that hospitals were not taking responsibility for follow-up of diagnostic test results at discharge from hospital.

Participants had strong feelings that discussion of medicines changes initiated by hospitals should be discussed with patients by secondary care staff at the point of discharge. GP participants did not feel they could rely on the hospital to do this, but noted a lack of time to do it themselves. National Institute for Health and Care Excellence (NICE) guidelines^19^ state that medicines reconciliations should be discussed with patients and carers. International attempts to impose requirement to discuss medicines reconciliation with patients in hospital have not always proved successful,^26,27^ although clear efforts are being made by secondary care to standardise the discharge experience.^16^ Direction of resources into primary care could allow GPs to perform the quality medicines reconciliation that they are perhaps best placed to deliver. The prevailing view of the participants was against delegation of prescribing responsibility to non-clinicians (mainly owing to safety and medicolegal concerns). The new 'pharmacist in general practice' role was clearly welcomed by participants as a resource in medicines reconciliation, but funding was considered a barrier,^28^ especially at smaller practices. The roll out of the pharmacists in general practice scheme may help to generate evidence, which might overcome these concerns.^29^

Participants were able to identify a broad range of error-producing conditions, but did not demonstrate a sophisticated understanding of how and why they occurred. They struggled to connect active failures on a particular discharge summary to broader latent failures within practice systems. Organisational factors^20^ were particularly hard to demonstrate as participants were almost entirely uncritical of their own practice systems. There was a reluctance by participants to discuss the role of the individual in the occurrence of error, perhaps because of the widely established concept that patient safety should not be a punitive field.^31,32^ Current political thinking on medical errors emphasises the need for the NHS to move away from a blame culture toward a learning culture.^33^ Systems need to be developed to mitigate human error, particularly to anticipate distraction from competing workload and time pressures (‘paperwork’ is GPs' fourth most likely job stressor).^34^ Cultural norms in general practice may make it challenging to promote interventions that prioritise dedicated time for paperwork. Responsibility for processing clinical documents in general practice is increasingly becoming an administrative role^35^ as promoted by the prime minister’s challenge fund,^22^ but as participants described, GPs would struggle to hand over control of discharge summaries to non-clinical staff for fear of missing out on important clinical information.

Participants described IT interventions to reduce error: streamlining existing task and diary systems and using Read-coded actions as failsafes for other systems. Interoperability of clinical systems both between secondary and primary care, and between practice clinical systems and EDMS, was considered highly desirable by the participants and is evidently achievable.^36^ IT systems for medicines reconciliation do exist in the US,^37^ but adaptation to the UK would need careful consideration and input from stakeholders. Most practices in the study were employing safety systems that they had ‘invented’ in-house to address particular local problems; sharing these examples using the Royal College of General Practitioners ‘bright ideas’ scheme,^37^ could facilitate spread of the best of these interventions.

### Implications for practice

Findings from the 'secondary care' theme suggest that there needs to be improvements in the quality of discharge summaries that are sent out from secondary care. Specific recommendations for discharge summary design, which are based on recurring subthemes present in the interview data, are included in Box 3. Hospitals should strive for standardisation and be reasonable in the requests made of primary care teams, particularly aiming to eliminate requests for follow-up of results of tests originating in secondary care.^38^ Box 3 describes suggestions made by participants in relation to the administrative team — such as protected time and/or workspace, and aiming for continuity of care while preserving timely transfer to doctors — and, for clinicians, suggestions about when and how decisions relating to discharge summaries should be documented. The participants were concerned about responsibility for medicines reconciliation, and this issue needs legal clarification as a follow-up to the Care Quality Commission report of 2011,^30^ with consideration of the role of the pharmacist both in primary and secondary care. Strategies captured in the 'safety improvement' theme indicate that there are implications for improvement of general practice systems (including IT) providing important opportunities for future research (see ‘wider systems redesign’, Box 3). More funding to alleviate pressure in general practice might allow practitioners to dedicate more time to tasks, such as carefully checking discharge summaries, thereby addressing the workload concerns of participants. Indeed, establishing administrative time for GPs (similar to that given to consultants as part of their Supporting Programmed Activities)^39^ might help embed this activity into the working week of GPs and help them to maintain personal responsibility for clinically important administrative work, which the participants desired. If processing discharge summaries becomes a task for administrative staff then resources need to be directed to set aside protected time for this activity in order to address the participants’ concerns on this matter.

This study has shown that general practice staff can identify where processes for managing discharge summaries might be improved by better documentation; development of IT; protection of workspace and time; and by enhanced communication with patients. The participants describe a need for consistency in the use of information in discharge summaries by secondary care, especially about results handling and medicines reconciliation. Such consistency and easy-to-understand standardisation should be assigned ‘Always Events’ status (a strategy to 'accelerate improvement efforts to enhance experiences of care for patients').^39^ More work is needed to characterise the safety interventions already in place in practices across the UK and to identify how actions can be processed most reliably and effectively in general practice.

## References

[1] Spencer RA, Spencer SEF, Rodgers S (2018). Processing of discharge summaries in general practice: a retrospective record review. Br J Gen Pract.

[2] National Institute for Health and Care Excellence (2015). Transition between inpatient hospital settings and community or care home settings for adults with social care needs. [NICE Clinical Guideline No G27]. https://www.nice.org.uk/guidance/ng27/chapter/Recommendations#discharge-from-hospital.

[3] Kings Fund Report (2014). Making our health and care systems fit for an ageing population. https://www.kingsfund.org.uk/publications/making-our-health-and-care-systems-fit-ageing-population.

[4] British Red Cross (2018). In and out of hospital. https://webcache.googleusercontent.com/search?q=cache:-QrL32AncwEJ:https://www.redcross.org.uk/-/media/documents/about-us/research-publications/health-social-care-and-support/in-and-out-of-hospital-report.pdf+&cd=1&hl=en&ct=clnk&gl=uk&client=firefox-b.

[5] Healthwatch (2017). What happens when people leave hospital and other care settings? Findings from the Healthwatch network. https://www.healthwatch.co.uk/sites/healthwatch.co.uk/files/20171004_what_happens_when_people_leave_care.pdf.

[6] Kripalani S, LeFevre F, Phillips CO (2007). Deficits in communication and information transfer between hospital-based and primary care physicians: implications for patient safety and continuity of care. JAMA.

[7] Williams H, Edwards A, Hibbert P (2015). Harms from discharge to primary care: mixed methods analysis of incident reports. Br J Gen Pract.

[8] Yemm R, Bhattacharya D, Wright D (2014). What constitutes a high quality discharge summary? A comparison between the views of secondary and primary care doctors. Int J Med Educ.

[9] Crowe S, Tully MP, Cantrill JA (2010). Information in general medical practices: the information processing model. Fam Pract.

[10] Royal College of General Practitioners (2017). Patient Safety Toolkit for General Practice. http://www.rcgp.org.uk/clinical-and-research/toolkits/patient-safety.aspx.

[11] Healthcare Improvement Scotland ihub (2019). Scottish patient safety programme: primary care. Safety across the interface. https://ihub.scot/spsp/primary-care/safety-across-the-interface/.

[12] Creswell JW, Plano Clark VL, Creswell JW, Plano Clark VL (2007). Examining preliminary considerations. Designing and conducting mixed methods research.

[13] Baxter P, Jack S (2008). Qualitative case study methodology: Study design and implementation for novice researchers. Qual Rep.

[14] Dean Franklin B, Shebl NA, Barber N (2012). Failure mode and effects analysis: too little for too much?. BMJ Qual Saf.

[15] Gale NK, Heath G, Cameron E (2013). Using the framework method for the analysis of qualitative data in multi-disciplinary health research. BMC Med Res Methodol.

[16] Mehta RL, Baxendale B, Roth K (2017). Assessing the impact of the introduction of an electronic hospital discharge system on the completeness and timeliness of discharge communication: a before and after study. BMC Health Serv Res.

[17] Institute for Healthcare Improvement (2019). Medication reconciliation to prevent adverse drug events. http://www.ihi.org/Topics/ADEsMedicationReconciliation/Pages/default.aspx.

[18] Kwan JL, Lo L, Sampson M (2013). Medication reconciliation during transitions of care as a patient safety strategy: a systematic review. Ann Intern Med.

[19] National Institute for Health and Care Excellence (2015). Medicines optimisation: the safe and effective use of medicines to enable the best possible outcomes [NICE Clinical Guideline No 5]. http://www.nice.org.uk/guidance/NG5/chapter/1-recommendations#medicines-related-communication-systems-when-patients-move-from-one-care-setting-to-another.

[20] Reason J (1990). The contribution of latent human failures to the breakdown of complex systems. Philos Trans R Soc B Bio Sci.

[21] Makeham MA, Stromer S, Bridges-Webb C (2008). Patient safety events reported in general practice: a taxonomy. BMJ Qual Saf.

[22] Salford CCG (2017). Salford integrated record. Sharing patient information locally. www.salfordccg.nhs.uk/download.cfm?doc=docm93jijm4n524.pdf&ver=680.

[23] NHS England (2016). Standards for the communication of patient diagnostic test results on discharge from hospital. https://www.england.nhs.uk/patientsafety/wp-content/uploads/sites/32/2016/03/discharge-standards-march-16.pdf.

[24] Göbel B, Zwart D, Hesselink G (2012). Stakeholder perspectives on handovers between hospital staff and general practitioners: an evaluation through the microsystems lens. BMJ Qual Saf.

[25] van Sluisveld N, Zegers M, Natsch S (2012). Medication reconciliation at hospital admission and discharge: insufficient knowledge, unclear task reallocation and lack of collaboration as major barriers to medication safety. BMC Health Serv Res.

[26] Greenwald JL, Halasyamani L, Greene J (2010). Making inpatient medication reconciliation patient centered, clinically relevant and implementable: A consensus statement on key principles and necessary first steps. J Hosp Med.

[27] Avery AJ (2017). Pharmacists working in general practice: can they help tackle the current workload crisis?. Br J Gen Pract.

[28] NHS England (2018). Clinical pharmacists in general practice. https://www.england.nhs.uk/gp/gpfv/workforce/building-the-general-practice-workforce/cp-gp/.

[29] Leape LL (1997). A systems analysis approach to medical error. Journal of Evaluation in Clinical Practice.

[30] British Medical Association NHS Employers (2011). A guide to consultant job planning. https://www.ed.ac.uk/files/imports/fileManager/national-job-planning-guide-consultants.pdf.

[31] Reason J (2000). Human error: models and management. BMJ.

[32] Department of Health and Social Care (2018). The plan to reduce medication errors: Q&A with Jeremy Hunt and Professor Dame Sally Davies. https://www.youtube.com/watch?v=VxfgjM_iZGg.

[33] Gibson J, Checkland K, Coleman A (2019). Eighth national GP worklife survey. http://www.population-health.manchester.ac.uk/healtheconomics/research/Reports/EighthNationalGPWorklifeSurveyreport/EighthNationalGPWorklifeSurveyreport.pdf.

[34] NHS Networks (2016). Medical assistants — workflow redirection in Brighton and Hove. https://www.networks.nhs.uk/nhs-networks/releasing-capacity-in-general-practice/documents/4-5-medical-assistants-workflow-redirection-in-brighton-and-hove/view.

[35] England NHS (2018). Training for reception and clerical staff. https://www.england.nhs.uk/gp/gpfv/redesign/gpdp/reception-clerical/.

[36] Schnipper JL, Liang CL, Hamann C (2011). Development of a tool within the electronic medical record to facilitate medication reconciliation after hospital discharge. J Am Med Inform Assoc.

[37] Royal College of General Practitioners (2017). Bright ideas. http://www.rcgp.org.uk/clinical-and-research/bright-ideas.aspx.

[38] Care Quality Commission (2009). National Study: Managing patient’s medications after discharge from hospital. http://webarchive.nationalarchives.gov.uk/20101122140156/http://www.cqc.org.uk/_db/_documents/Managing_patients_medicines_after_discharge_from_hospital.pdf.

[39] Institute for Healthcare Improvement for NHS England (2018). Always Events Toolkit. https://webcache.googleusercontent.com/search?q=cache:XEE9mau8neEJ:https://www.england.nhs.uk/wp-content/uploads/2016/12/always-events-toolkit-v6.pdf+&cd=1&hl=en&ct=clnk&gl=uk&client=firefox-b-ab.

